# Asymptomatic Periaortitis Following Endovascular Abdominal Aortic Repair: A Case Report of Spontaneous Resolution with Recurrence and Reactivation during Treatment

**DOI:** 10.3400/avd.cr.26-00013

**Published:** 2026-07-08

**Authors:** Masahiro Aiba, Makoto Hamaishi

**Affiliations:** Department of Cardiovascular Surgery, Totsuka Kyoritsu Second Hospital, Yokohama, Kanagawa, Japan

**Keywords:** periaortitis, endovascular aortic repair

## Abstract

A patient who underwent endovascular abdominal aortic repair for a non-inflammatory abdominal aortic aneurysm developed asymptomatic periaortitis 2.5 years postoperatively. Conservative management resulted in aneurysm shrinkage and reduced inflammation. However, at 6 years postoperatively, tumor-like soft tissue shadows appeared around the aorta and the aneurysm diameter re-expanded. This was diagnosed as a recurrence and worsening. Prednisolone was initiated, leading to reductions in both aneurysm size and the mass-like shadow size. When the dosage was decreased, inflammation flared up again. The medication dose was then increased. Gradual dose reduction was subsequently attempted and treatment was successfully discontinued without recurrence.

## Introduction

Periaortitis following endovascular aortic repair (EVAR) for a non-inflammatory abdominal aortic aneurysm (AAA) is a rare complication. Although several cases have been reported, the etiology and optimal treatment remain poorly understood. We report a case that developed clinically asymptomatically 2.5 years after EVAR, with improvement initially seen with observation alone, but relapse and worsening 6 years postoperatively. Treatment was initiated, but recurrence developed during therapy. We present this case along with a review of the literature.

## Case Report

A 58-year-old man was admitted to the gastroenterology department with a suspected gastric ulcer. Plain computed tomography (CT) of the abdomen revealed an AAA. His medical history included cardiac intervention due to acute myocardial infarction at 44 years of age, chest trauma at 50 years of age, hypertension, and diabetes. In addition, he reported no drug or metal allergies. Abdominal contrast-enhanced CT revealed a fusiform aneurysm with a maximum diameter of 52 mm below the origin of the renal arteries; no intimal thrombus was present. No findings indicative of periarterial inflammation were observed (**Fig. 1A**). Preoperative blood analysis showed a normal white blood cell (WBC) count of 5080/μL (normal, 3800–9300/μL), a normal C-reactive protein (CRP) level of 0.22 mg/dL (normal, 0.00–0.30 mg/dL), and normal renal function with a creatinine level of 0.66 mg/dL (normal, 0.40–1.10 mg/dL). Echocardiography revealed an ejection fraction of 47% (normal, 50%–80%).

**Fig. 1:**
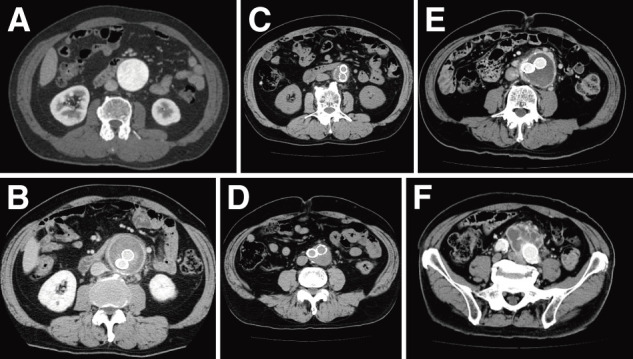
(**A**) Preoperative abdominal contrast-enhanced CT. A fusiform aneurysm with a maximum diameter of 52 mm was identified below the origin of the renal artery. No signs of periarterial inflammation were observed. (**B**) Contrast-enhanced CT at 2.5 years postoperatively. The aneurysm diameter had enlarged to 58.0 mm. Thickening of the aneurysm wall, an ill-defined area of abnormal absorption around the artery, and multiple enlarged lymph nodes were observed. (**C**, **D**) Plain CT at 5 years postoperatively. The aneurysm diameter rapidly decreased to 44.5 mm, with reduced aneurysm wall thickening and surrounding inflammatory changes. (**E**, **F**) Contrast-enhanced CT at 6 years postoperatively. Re-expansion of the aneurysm diameter (50.8 mm) was observed, and in addition to thickening of the aneurysm wall, a tumor-like soft tissue shadow extending anteriorly at the aortic bifurcation was noted. CT: computed tomography

### Surgery

EVAR was performed using a bifurcated stent graft (Gore Excluder AAA endoprosthesis; W. L. Gore & Associates, Flagstaff, AZ, USA) under general anesthesia. The patient was discharged on postoperative day 7 without surgical complications. Contrast-enhanced CT performed before discharge revealed a type II endoleak originating from the lumbar artery.

### Initial findings

CT at 1 year postoperatively showed that the aneurysm diameter had decreased to 50.5 mm. However, contrast-enhanced CT at 2.5 years postoperatively revealed that the aneurysm diameter had enlarged to 58.0 mm, and showed thickened aneurysm walls, poorly defined areas of abnormal absorption around the aorta, and multiple enlarged lymph nodes, leading to a diagnosis of inflammatory changes (**Fig. 1B**). In addition, the type II endoleak was observed to be confined to the posterior wall of the right limb of the stent graft. Blood testing at this time showed a slight elevation in inflammatory markers (CRP 2.01 mg/dL), but the patient reported no symptoms and showed no signs of illness, so no treatment was administered. Digital subtraction angiography revealed blood flow into the aneurysm via the left iliac artery through the fourth lumbar artery. However, embolization was deemed impossible due to stenosis at the origin of the left iliac artery and was therefore not performed.

### Spontaneous regression

As no symptoms developed thereafter, follow-up observation was continued. At 5 years postoperatively, the aneurysm diameter had rapidly decreased to 44.5 mm, with reductions in wall thickening and surrounding inflammatory changes (**Fig. 1C** and **1D**).

### Recurrence and worsening

However, after another year (at 6 years postoperatively), contrast-enhanced CT revealed re-expansion of the aneurysm diameter to 50.8 mm. In addition to thickening of the aneurysm wall, a tumor-like soft tissue shadow was noted extending anteriorly at the aortic bifurcation (**Fig. 1E** and **1F**). The patient still reported no subjective symptoms such as fever or pain, but had lost 5 kg in weight over the preceding 5 months. Blood tests showed: WBC, 5270/μL; neutrophils, 61.2% (both within normal ranges); CRP, 3.66 mg/dL; erythrocyte sedimentation rate (1-h value), 46 mm (normal, 1–15 mm); gamma globulin, 26.3% (normal, 10.2%–20.4%); complement level, 58 U/mL (normal, 30–45 U/mL); negative results for all antinuclear antibodies; and immunoglobulin (Ig)G4, 41 mg/dL (normal, 4–108 mg/dL). Positron emission tomography-CT (PET-CT, ^18^F-fluorodeoxyglucose [FDG]-labeled) revealed uptake in the abdominal aortic wall and adjacent soft tissues, with marked enhancement at the aortic bifurcation and ventral to the common iliac arteries. Recurrence and worsening of periaortic inflammation following EVAR were diagnosed (**Fig. 2A**).

**Fig. 2:**
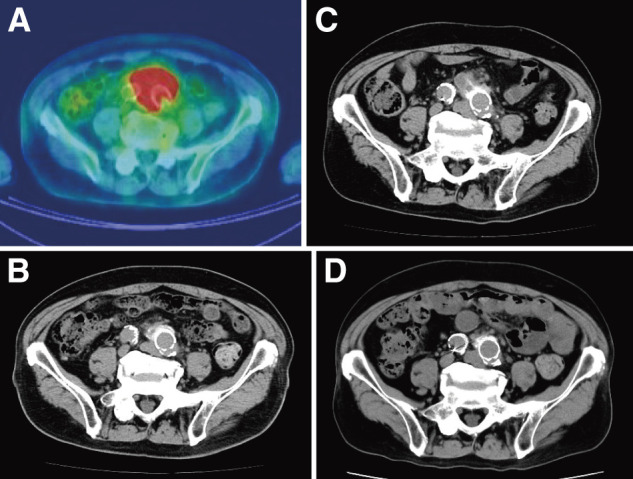
(**A**) PET-CT (FDG). Accumulation was observed in the abdominal aortic wall and adjacent soft tissues, with particularly intense accumulation at the aortic bifurcation and on the ventral side of the common iliac arteries. (**B**) Plain CT 1 month after treatment. The tumor-like soft tissue shadow decreased in size, and the aneurysm diameter also decreased. (**C**) Plain CT 10 months after treatment initiation. There is a recurrence of the tumor-like soft tissue shadow, with re-expansion of the aneurysm diameter. (**D**) Plain CT 10 years postoperatively, 5 years after treatment initiation. No change in aneurysm diameter; no recurrence of the tumor-like soft tissue shadow at the bifurcation. FDG: ^18^F-fluorodeoxyglucose; PET-CT: positron emission tomography-computed tomography; CT: computed tomography

### Treatment initiation

Although the patient remained clinically asymptomatic, treatment was considered to be indicated because inflammation had spread to the retroperitoneal space, and prednisolone (PSL) was initiated at 60 mg/day. CT at 1 month after starting treatment showed a reduction in the tumor-like soft tissue shadow, with the aneurysm diameter decreasing to 49.4 mm (**Fig. 2B**). Further, at the 3-month follow-up, the aneurysm diameter had decreased further. PSL was therefore gradually tapered to 5 mg/day over a 6-month period.

### Relapse during tapering

However, CT at 10 months after treatment initiation showed recurrence of the tumor-like soft tissue shadow, with the tumor diameter also re-expanding to 52.5 mm (**Fig. 2C**). The dose of PSL was therefore increased to 30 mg/day. After confirming no enlargement of the tumor-like shadow or aneurysm on CT, PSL was gradually tapered again. CT at 3 years after treatment showed the aneurysm diameter had decreased to 48.6 mm and the tumor-like shadow at the bifurcation had also diminished, so PSL was finally discontinued (**Fig. 2D**).

### Final remission

Subsequent CT 10 years after surgery showed no recurrence of the tumor-like shadow at the bifurcation or change in aneurysm diameter.

## Discussion

Periaortitis following EVAR is a rare complication characterized by chronic, specific retroperitoneal inflammation occurring after EVAR for non-inflammatory AAA. The mechanisms remain incompletely understood, but autoallergic or immune reactions to graft or stent materials, or degeneration of atherosclerotic plaques or intraluminal thrombi, are suspected to be involved.^1–4)^ The diagnosis can be suspected based on clinical symptoms and imaging studies, but a differential diagnosis is necessary to distinguish retroperitoneal inflammation from inflammatory aneurysms, retroperitoneal fibrosis, IgG4-related disease, and various conditions causing chronic aortic inflammation, such as aortitis syndromes or giant cell arteritis affecting large vessels, infections, and tumors (lymphoma or sarcoma).

Preoperative imaging in the present case did not reveal any evidence of inflammation in the aortic aneurysm wall or surroundings. The patient did not report any subjective symptoms when inflammatory changes first appeared, so no detailed examinations were performed at that time. However, immunoserological testing conducted at both recurrence and the worsening 6 years postoperatively showed negative results for all antinuclear antibodies and normal IgG4 levels. Histological examination is considered valuable for diagnosing autoimmune diseases such as IgG4-related disease, but was deemed too invasive in this asymptomatic case and therefore was not performed. While tissue biopsy has been reported, immunological testing was not described, and the condition was only diagnosed as aseptic inflammation.^5)^ Further, infection or tumor was suspected based on imaging, but each was ruled out by systemic findings, laboratory results, and PET-CT. The overall diagnosis was periaortitis following EVAR.

Treatment with PSL at an initial dose of 1.0 mg/kg was selected based on past case reports and the Vascular Inflammatory Syndrome Treatment Guidelines (2017 revised edition), and the dose was gradually tapered. Although the patient did not have any subjective symptoms and showed no increases in inflammatory markers, imaging at 10 months revealed recurrence. The PSL dose was therefore increased, resulting in improvement. After further cautious tapering, PSL was finally discontinued 3 years after initiation.

Since Simons et al.’s first reported periaortitis following EVAR in 2002,^1)^ we have identified 11 reports in the foreign-language literature to date.^1–10)^ The age, sex, preoperative aneurysm size, stent graft used, onset period, diagnosis, symptoms, accompanying findings, presence or absence of endoleak and enlargement, and treatment drugs for previously reported cases are shown in **Table 1**. Various stent grafts were used, but all stents were nitinol. Mean time to onset was 9.6 months following EVAR, with the majority (73%) occurring within 1 year. In all previously reported cases, patients presented with clinical symptoms, including pain such as abdominal or back pain, fever, and fatigue. Concurrent lesions included ureteral obstruction or hydronephrosis in 5 cases, and inflammation had progressed to the inferior vena cava in 2 cases. Conversely, only 1 case each showed enlargement or development of endoleak. Treatment in early cases was either with tamoxifen alone or in combination with PSL. However, recent cases more frequently used PSL alone. While some cases continued PSL or tamoxifen therapy, most were able to discontinue treatment within 6–12 months.

**Table 1:** Previous cases and our own case of periaortitis following EVAR

	Published	Age (years)	Sex	AAA size	Stent graft	Onset after EVAR	Symptoms	Ureteric obstruction and treatment
Simons^1)^	2002	59	M	n.s.	Talent	17 months	Left-sided abdominal pain	Left ureter, nephrectomy
Jetty^2)^	2004	76	M	54 mm	AneuRx	5 months	Fatigue, anuria	Bilateral ureter, HD, and stent
Brouw^3)^ (1)	2007	60	M	n.s.	n.s.	36 months	Abdominal pain, back pain, fatigue, fever	No
Brouw^3)^ (2)	2007	72	M	n.s.	n.s.	3 months	Back pain, fatigue	No
Vijaynagar^4)^	2011	63	M	55 mm	Cook	9 months	Left loin pain	Left ureter, stenting
Peters^5)^	2013	81	M	n.s.	n.s.	12 months	Loss of appetite, nausea, weight loss, back pain	Right hydronephrosis, conservative
Mansour^6)^	2015	67	M	53 mm	Excluder	3 weeks	Lower limb heaviness, back pain	No
Frech^7)^	2015	70	n.s.	65 mm	Endurant	3 months	Back pain	Left ureter, conservative
Taguchi^8)^	2016	78	M	n.s.	n.s.	15 months	Abdominal pain	No
Trinder^9)^	2019	64	M	66 mm	Excluder	2 months	Abdominal pain, night sweat	No
Samura^10)^	2019	72	M	68 mm	Excluder	3 months	Fever, back pain	No
This case	2026	58	M	52 mm	Excluder	30 months	No symptoms	No
	Other complications	PET-CT		Endoleak		Expansion	PSL (initial dose, period of use)	Tamoxifen
Simons^1)^				No		No	No	10 mg twice daily
Jetty^2)^	IVC involvement			No		No	60 mg/day, 12 months	10 mg twice daily (continued)
Brouw^3)^ (1)							40–60 mg/day, 6 weeks and 5–10 mg, 6–12 months	No
Brouw^3)^ (2)							40–60 mg/day, 6 weeks and 5–10 mg, 6–12 months	No
Vijaynagar^4)^				No		No	90 mg/day, 12 weeks	20 mg daily (continued)
Peters^5)^		Yes				No	60 mg/day, 9 months and 7.5 mg/day (continued)	No
Mansour^6)^	IVC involvement						16 mg/day, 6 months	No
Frech^7)^				No			80 mg/day; maintenance 4 mg/day, 12 months	No
Taguchi^8)^				No		No	High doses	No
Trinder^9)^		Yes		Yes (IMA)		Yes (74 mm)	75 mg/day, 5 months	No
Samura^10)^						No	30 mg/day, 2 weeks and reduced to 5 mg (continued)	No
This case		Yes		Yes (lumbar)		Yes (59.0 mm)	60 mg/day, and reduced to 5 mg, 6 months, but recurred	No

AAA: abdominal aortic aneurysm; EVAR: endovascular aortic repair; HD: hemodialysis; IMA: internal mammary artery; IVC: inferior vena cava; M: male; PET-CT: positron emission tomography-computed tomography; PSL: prednisolone; n.s.: not specified; blank space: not reported

Compared to previous reports, the distinguishing features of this case were the clinically asymptomatic presentation and the pattern of alternating improvements and recurrences. Although some weight loss was observed during periods of relapse or worsening, the patient never reported any subjective symptoms. Further, we have consistently observed the patient since before surgery, noting no changes in quality of life or function. The reason for the consistent absence of subjective symptoms throughout the course of this case remains unclear, but may be related to a lack of involvement of surrounding organs. The reasons for spontaneous resolution and recurrence are also unknown. Similar changes have been reported in the past (recurrence rate, 17.9%, including this case),^10)^ but no previous cases have exhibited repeated enlargement or reduction of the aneurysm during both onset and improvement, as seen in this case.

Although type II endoleak was present immediately after surgery in this case, the blood flow associated with the endoleak may have influenced the material changes in the stent graft or the state of the thrombus, potentially contributing to the onset. At the time of onset, pressure from the endoleak was considered to have acted on the aortic wall weakened by inflammation, causing an expansion of the aneurysm diameter. Similarly, a case reported by Trinder et al. in which the aneurysm enlarged after onset also showed type II endoleak at that time.^9)^ However, the reason for spontaneous remission in the present case is unclear. Although the causes of recurrences and exacerbations remain unclear, imaging revealed that the legs of the stent graft were in close contact with the shrunken aneurysmal wall. Atherosclerotic plaques arising from injury or breach of the aortic intima have been suggested to induce localized chronic inflammation.^4)^ Pulsatile movement of the stent graft may have caused damage to the previously inflamed aortic wall, leading to localized recurrence of inflammation.

No protocol has been established regarding the dosage or duration of PSL administration for treating periaortitis following EVAR, and treatment is often based on the approach for inflammatory aneurysms or autoimmunity-related aortitis. However, in cases of aortitis involving an autoimmune mechanism, some patients experienced recurrence during PSL tapering or after treatment, as seen in this case. Further, while there have been cases in which medication was successfully discontinued, other cases have required maintenance therapy even after remission. Given the potential for various mechanisms underlying the development of periaortitis following EVAR, long-term maintenance therapy with PSL may be necessary in cases with recurrent flare-ups or strongly suspected autoimmune involvement.

## Conclusion

We encountered a patient who developed periaortitis without symptoms 2.5 years following EVAR for non-inflammatory AAA. The inflammation initially improved spontaneously, but relapsed and worsened 6 years postoperatively. Treatment with PSL was initiated, but a relapse occurred during dose reduction.

Follow-up should be conducted with the awareness that periaortitis can develop even without clinical symptoms following EVAR. Further, while PSL is considered effective for treatment, the possibility of recurrence during tapering must be considered.
